# Neutrophil extracellular traps in acute coronary syndrome

**DOI:** 10.1186/s12950-023-00344-z

**Published:** 2023-05-10

**Authors:** Yawen Wu, Shilin Wei, Xiangyang Wu, Yongnan Li, Xue Han

**Affiliations:** 1 Department of Cardiac Surgery, Lanzhou University Second Hospital, Lanzhou University, No. 80 Cuiyingmen, Chengguan District, Lanzhou, 730030 China; 2 Department of Thoracic Surgery, Lanzhou University Second Hospital, Lanzhou University, Lanzhou, China; 3 Second Clinical Medical College, Lanzhou University, Lanzhou, China

**Keywords:** Neutrophil extracellular traps, Acute coronary syndrome, Atherosclerosis, Myocardial infarction, Heart failure

## Abstract

Acute coronary syndrome (ACS) is a group of clinical syndromes caused by acute myocardial ischemia, which can cause heart failure, arrhythmia and even sudden death. It is the major cause of disability and death worldwide. Neutrophil extracellular traps (NETs) are reticular structures released by neutrophils activation and have various biological functions. NETs are closely related to the occurrence and development of ACS and also the subsequent damage after myocardial infarction. The mechanisms are complex and interdependent on various pathways, which require further exploration. This article reviewed the role and mechanism of NETs in ACS, thereby providing a valuable reference for the diagnosis and clinical treatment of ACS.

## Introduction

Acute coronary syndrome (ACS) is an internationally recognized acute cardiovascular disease characterized by rapid onset and progression with a high mortality rate [1]. The pathogenesis of ACS is divided into three stages. In the early stage, the disease is mainly characterized by the rupture or invasion of coronary atherosclerotic plaque, followed by rational thrombosis. During the middle and late stages, ischemic myocardium necrosis in the diseased coronary arteries leads to myocardial infarction (MI) and, eventually, heart failure or even death. Recent studies have confirmed that ACS is the leading mortality-causing clinical outcome in patients with cardiac diseases [2]. Therefore, it is vital to explore practical strategies for the management of ACS and elucidate the inducers and mechanisms of ACS, thereby improving the prognosis of patients.

Neutrophils, immune cells in the innate immune system, are the body’s first line of defence against pathogen invasion, inducing various pathological processes such as the induction of a series of responses to inflammatory stimuli. Recently, neutrophils have been reported to function through the NETosis defence mechanism, which releases a reticulum called neutrophil extracellular traps (NETs) [3]. The NETs comprise DNA and histones released by activated neutrophils, which play a role in the neutrophil-mediated intrinsic immune [4]. The NETs were initially considered as effector proteins that protect the human body against pathogens, immobilizing pathogens and exposing them to high local concentrations of a bactericidal environment [5]. This review systematically analyzed and summarized the mechanism of NETs in ACS. First, we introduce the structure and function of NETs. Second, we describe the impact of NETs on ACS from the point of view of atherosclerosis, MI and heart failure. Finally, we discuss the role of NETs as a disease prediction marker of ACS, which could aid in its clinical intervention and treatment.

## Introduction to NETs

### Structure and function of NETs

**Fig. 1:**
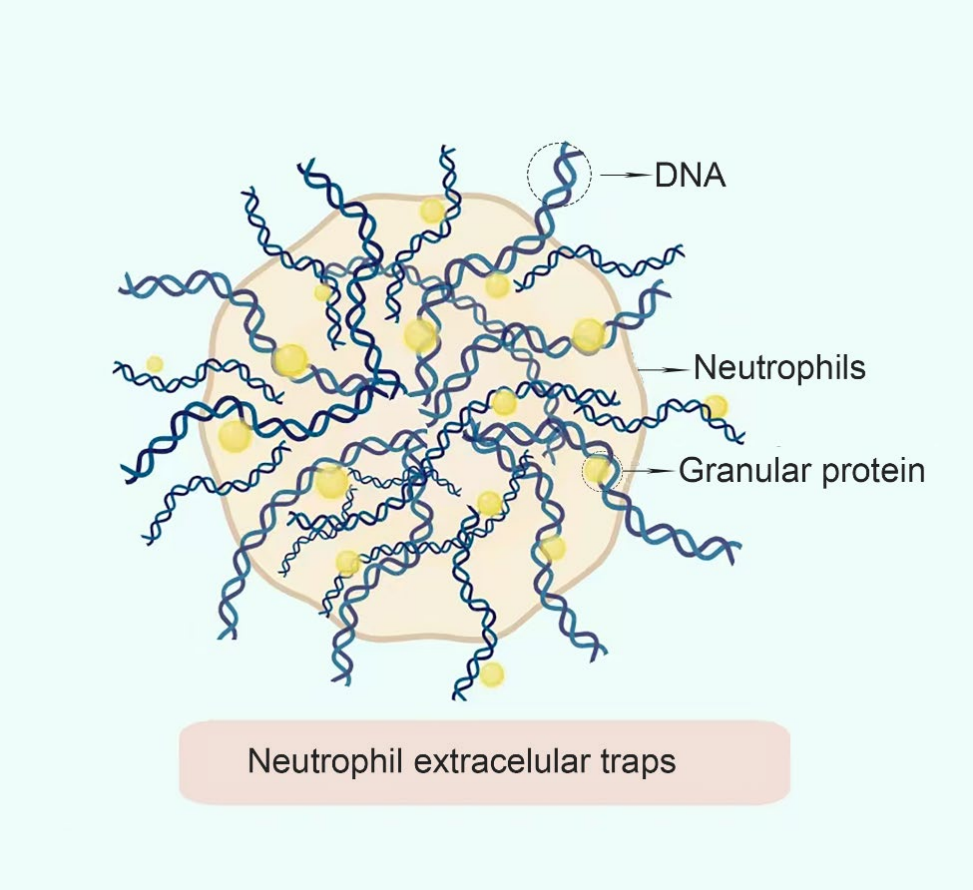
Structure of neutrophil extracellular traps (NETs). NETs are a reticular structure composed of DNA and granular proteins secreted by activated neutrophils. They regular the capturing and killing of extracellular pathogens and exert a protective role during the antibacterial defense

In 2004, Brinkmann et al. [6] first observed the structure of NETs on stimulating neutrophils with phenyl propyl acetate myristate (PMA), lipopolysaccharide (LPS) and interleukin 8 (IL-8). The core components of NETs are depolymerized DNA, histones, particle components and other related proteins that are assembled on a chromatin scaffold [7], eventually forming a lethal environment that prevents the participation of neutrophils in the body’s immune defence system, which consequently prevents the invasion of pathogens and destruction of the invaded pathogens. Studies [8, 9] report that DNase can cleave NETs, degrade their essential skeletal DNA and inhibit the extracellular bactericidal function of neutrophils. Therefore, the DNA backbone and histones are considered essential and indispensable components of NETs. Furthermore, factors that induce NETs are extracellular pathogens, viruses [10], fungi [11], cholesterol and urate crystals, lipids, activated platelets [12] and complements such as C5a (Fig. 1).

### Formation of NETs

The production of NETs and NETosis, which have potential roles in various diseases, is considered an effective weapon in sterile inflammation [13]. NETs have long been considered a critical pathogen barrier that induces microbial death by binding to microorganisms, inhibiting their dissemination and maintaining a locally high-concentration antimicrobial environment [14]. Moreover, NETs are also crucial in non-pathogenic infections and have been associated with several cardiovascular diseases and corresponding risk factors [15]. The release of NETs is premised on the rupture of the plasma membrane, wherein the activation of neutrophils leads to changes in their morphological structure, resulting in chromatin fragmentation, nuclear rupture and ultimately the release of NETs [16]. The process of neutrophils secreting NETs is known as NETosis, which is also known as the inflammatory death mode of neutrophils. NETosis involves multiple signal pathways and molecular mechanisms; however, studies on its formation mechanism at home and abroad are scarce.

**Fig. 2:**
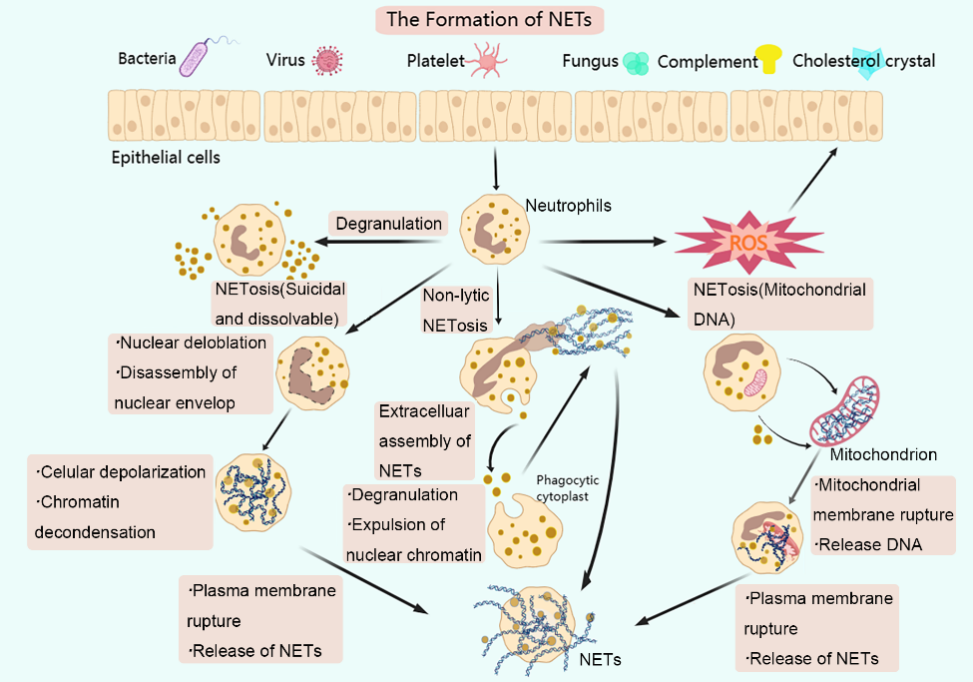
The formation of neutrophil extracellular traps (NETs). Bacteria, viruses, fungi, cholesterol and urate crystals, lipids, activated platelets and complements can induce neutrophil activation and produce NETs through three secretory modes: suicidal, non-lytic and mitochondrial DNA release. The process of neutrophils secreting NETs is called NETosis.

Based on current studies, NETs are secreted via three pathways. (1) Suicidal and lytic NETosis [5, 17]: neutrophils upon stimulation by PMA or IL-8, activate protein kinase C and RAF-mitogen-activated protein kinase-extracellular signal-regulated kinase pathways in the cell. This process lasts for a long time, usually, 2 to 4 h. PMA, LPS and various bacterial-related activation stimulate the RAF-MEK-ERK pathway through the NADPH oxidase 2 (NOX2) complex, producing reactive oxygen species (ROS) that act as secondary messengers to promote the detachment of the nuclear envelope and finally leading to the rupture of the plasma membrane and release of decondensed chromatin. (2) Non-lytic NETosis [18]: this form occurs for a shorter period, generally within 30–60 min and is induced by the activation of neutrophils mediated by toll-like receptors. Notably, it is independent of NADPH oxidase activation. Moreover, cell function can still be preserved as it does not disrupt the nuclear envelope and cell membrane. Herein, the release of nuclear material is mainly achieved by vesicular export. (3) Mitochondrial DNA releases NETs [19]: this method was recently discovered by Yousefi et al. This process is also dependent on the ROS pathway; however, the released DNA originates from the mitochondria rather than the nucleus. Notably, this process can last for 15 min (Fig. 2).

However, controversies and inconsistencies over describing the formation mechanism of NETs with respect to NETosis remain as NETosis initially only represents the release of soluble NETs and can be distinguished from cell death and lysis with respect to this aspect [20]. However, numerous studies have shown that not all NETs formation pathways lead to cell lysis and death. The NETs formation is a complex process regulated by multiple pathways, with differences in the formation mechanism of NETs under varying stimulatory substances. However, the exact mechanism of NETs formation remains unclear and requires further exploration.

### Biomarkers of NETs and detection methods

Assessing the ability of neutrophils to form NETs in peripheral blood would be a promising method for predicting ACS. Several possible methods for the detection of NETs will be discussed below.

#### In situ or in vivo detection methods

To evaluate the formation ability of NETs, it is necessary to evaluate peripheral blood neutrophils, which can be non-invasive detected through characteristic in situ or in vivo detection methods: (1) In vivo imaging techniques: In vivo imaging refers to the application of imaging methods at the cellular and molecular levels in the living state for the qualitative and quantitative analysis of biological processes and temporal. In 2006, Buchanan et al. used DNA embedding dyes to demonstrate a significant correlation between DNA enzymes, NETs degradation, and pathogenicity [21]. Fuchs et al. used live cell imaging combined with phase contrast, survival markers, death markers, and histone staining to find that PMA activates neutrophils and releases NETs by neutrophils are accompanied by their death [22, 23]. Imaging techniques of different living cells have been widely used to detect a variety of diseases; (2) In vivo microscopy techniques: In vivo, microscopy can visualize protein activity, gene expression, cellular transport, cell-cell/cell-microenvironment interactions, and a range of physiological responses to stimuli in vivo, facilitating dynamic 3D in vivo cellular-level imaging of a range of biological processes in living animals. Clark et al. observed NETs produced by activated platelets in the liver sinusoids by in vivo microscopy. As living organisms continue to deepen, various types of microscopes are used to detect the formation of NETs in different tissues and organs [24].

#### In vitro assays

There is no reliable and standardized method to measure the formation of NETs in vivo, and an in vitro neutrophil function test may be a potential option. As used in COVID-19, neutrophils can be isolated from peripheral blood and then stimulated with inflammatory triggers used to induce the formation of NETs, and the resulting NETs can then be quantified. Some in vitro assays for NETs are presented next [25].

##### **Co-localization of neutrophil derived proteins and extracellular DNA**

Co-localization of neutrophil-derived proteins and extracellular DNA is a standard method for in vitro detection of NETs, where neutrophils are inoculated on slides, incubated for several hours with or without stimulation, fixed, and then immunostained for neutrophil-derived proteins and DNA, and co-localization of proteins and DNA is indicative of the presence of NETs [26, 27]. This method is easy to perform but requires certain precautions to permeabilize the plasma membrane while fixing the cells. Usually, these reagents induce the formation of artificial NETs, which may impact the results. In addition, the method lacks objectivity, and the investigator needs to autonomously assess whether the derived proteins and DNA are distributed inside or outside the neutrophils, with a particular subjectivity [28, 29].

##### **Flow cytometry detection**

The following flow cytometry assays are commonly used − (1) Indirect immunofluorescence assay: cfDNA is the essential backbone of NETs, and DNA staining can be used to visualize NETs. Antibodies to two significant components of NETs, citrullinated histone (CitH3) and myeloperoxidase (MPO), are combined with DNA dyes and detected by an indirect immunofluorescence method [30]. Since this method is based on CitH3 antibody staining, it cannot be used in CitH3-deficient mice even though their NETs formation capacity is still present [31]; (2) Image-based flow cytometry detection: in a characteristic swelling assay on neutrophil nuclei, Zhao et al. used a Ficoll density gradient to isolate neutrophils from whole blood samples granulocytes, which were then separated from the erythrocyte layer by dextran sedimentation [32]. After effective stimulation, neutrophils were fixed in a 2% PFA solution containing 1:1000 dilution of Hoechst, nuclear labeling was performed, and MPO staining was performed. The results showed an approximately 3-fold increase in the mean nuclear area of neutrophils compared to unstimulated cells, confirming that NETs can be detected using multispectral imaging flow cytometry; (3) High-speed multispectral imaging flow cytometry [32]: effective cellular figuration, i.e., volume, morphology, and associated sub localization of cellular structures. In this approach, it is possible to distinguish between lysogenic and non-lysogenic NETs, and this approach also has the drawback that the imaging system only shows NETs formed in the immediate state and cannot detect NETs subsequently released from cells that have started lysis. Therefore, several flow cytometric techniques allow for specific, quantitative, and rapid detection of neutrophil morphology, providing feasibility for further studies of NETs as bioindicators in clinical medicine [30, 32].

##### **Immunostaining analysis of guanine histones**

The presence of guanosine histones specifically indicates that PAD4 mediates the induction of NETs. PAD4 is expressed mainly in hematopoietic cells and induces guanosylation of arginine, which de-agglutinates chromatin from activated neutrophils and forms NETs. The presence of guanosine histones, as determined by immunostaining, is evidence of NET formation in vitro and in vivo [26]. However, it has been shown that the involvement of PAD4 in NET formation depends on the nature of the stimulus; therefore, PAD4 is controversial in the NET formation process. Moreover, the method is only applicable to PAD4-dependent NETosis [33, 34].

##### **Enzyme-linked immunosorbent assay**

The basic principle of enzyme-linked immunosorbent assay (ELISA) is that an enzyme couple with an antibody or antigen to form a complex, and when the antigen-antibody binds explicitly, the complex can catalyze the conversion of a colorless substrate molecule into an easily detectable substance, and the change in the substrate signal can determine whether the immune reaction has occurred and thus analyze the concentration of the target substance [35]. This method detects CitH3, MPO, and DNA, the main components of NETs, to indirectly prove the presence of NETs and to quantify them [35, 36].

In summary, NETs formation is a specific manifestation of neutrophils after responding to infection and inflammatory reactions. Various NETs detection techniques have been implemented in recent years to visualize NETs. However, the current technical means of visualization still have different degrees of defects. In addition, for in vitro NETs testing, although promising results have been shown, challenges remain and optimization of experimental protocols and measurement techniques may be required. Thus, even though neutrophil function assays, especially in vitro assays, have great potential in predicting ACS episodes, more studies are needed to determine their validity and utility in the clinical setting.

## NETs in ACS

### NETs and atherosclerosis and thrombosis

Atherosclerotic lesions start from the intima, accompanied by arterial medial degeneration and calcification and ultimately lead to the thickening and hardening of the arterial wall and narrowing of the vessel lumen, which is the pre-lesion stage of ACS. Megen et al. first reported [37] that neutrophils and NETs are present in atherosclerotic lesions in mice and humans. Additionally, Kartika et al. [38] reported that ACS could be caused by the rupture of the fibrous cap of atherosclerotic plaques, plaque erosion, or intraplaque hemorrhage in patients. Moreover, at the thrombus, hemorrhage and thrombus-plaque interface, neutrophils and NETs were abundantly present in all types of complex plaques, indicating the potential role of NETs in the formation of atherosclerosis. Furthermore, NETs can damage the endothelium through the combined action of IL-1 and cathepsin G, which promotes the endothelial expression of ICAM-1, VCAM-1 and tissue factors and is associated with thrombus formation owing to the erosion of the atherosclerotic plaque surface [39]. Silvestre et al. [40] observed that the externalized histone H4 on NETs could lead to the lysis of smooth muscle cells, wherein the plaques become fragile and can be easily sloughed off. Many studies have revealed the mechanism by which NETs accelerate thrombosis. Döring Y et al. [41] reported that interacting neutrophils and platelets at the site of plaque rupture can promote NETs formation and stimulate active tissue factors to accelerate thrombus formation. Nicoletta et al. [42, 43] reported that, in APOE-deficient mice. NETs formed in the early stage of atherosclerotic lesions. Meanwhile, relevant experimental data also suggest that NETs can promote erosive effects on the plaque surface, thereby exposing thrombotic material in the plaque to locally high concentrations of NETs and accelerating thrombus formation [44]. Additionally, cholesterol crystals formed during atherosclerotic lesions could also stimulate the release of NETs, which can induce macrophages to secrete cellular inflammatory factors and thereby promote the migration of neutrophils to inflammatory sites [45]. Thus, the above findings indicate that NETs play an essential role in the inflammatory response.

NETs are significant contributors to the formation of pathological thrombi. In 2010, Fuchs et al. [46] demonstrated that NETs provided a physical scaffold for thrombus growth by binding to platelets and erythrocytes. The primary mechanism is speculated to be that NETs can promote the adhesion and aggregation of platelets and the binding of red blood cells, consequently promoting the formation of blood clots. Conversely, NETs can promote thrombin generation by activating platelets and coagulation factors XI and XII [47], which activate intrinsic and extrinsic coagulation pathways and accelerate thrombus formation. However, the procoagulant effect of the NETs complex does not compare to that of DNA or histone components alone [17]. The process by which histones and DNA are tightly bound to nucleosomes could reduce their ability to interact with the coagulation system, thus making them more potent procoagulants. However, studies report that histones cannot directly drive the coagulation cascade but can induce thrombin generation by activating platelets [48, 49].

**Fig. 3:**
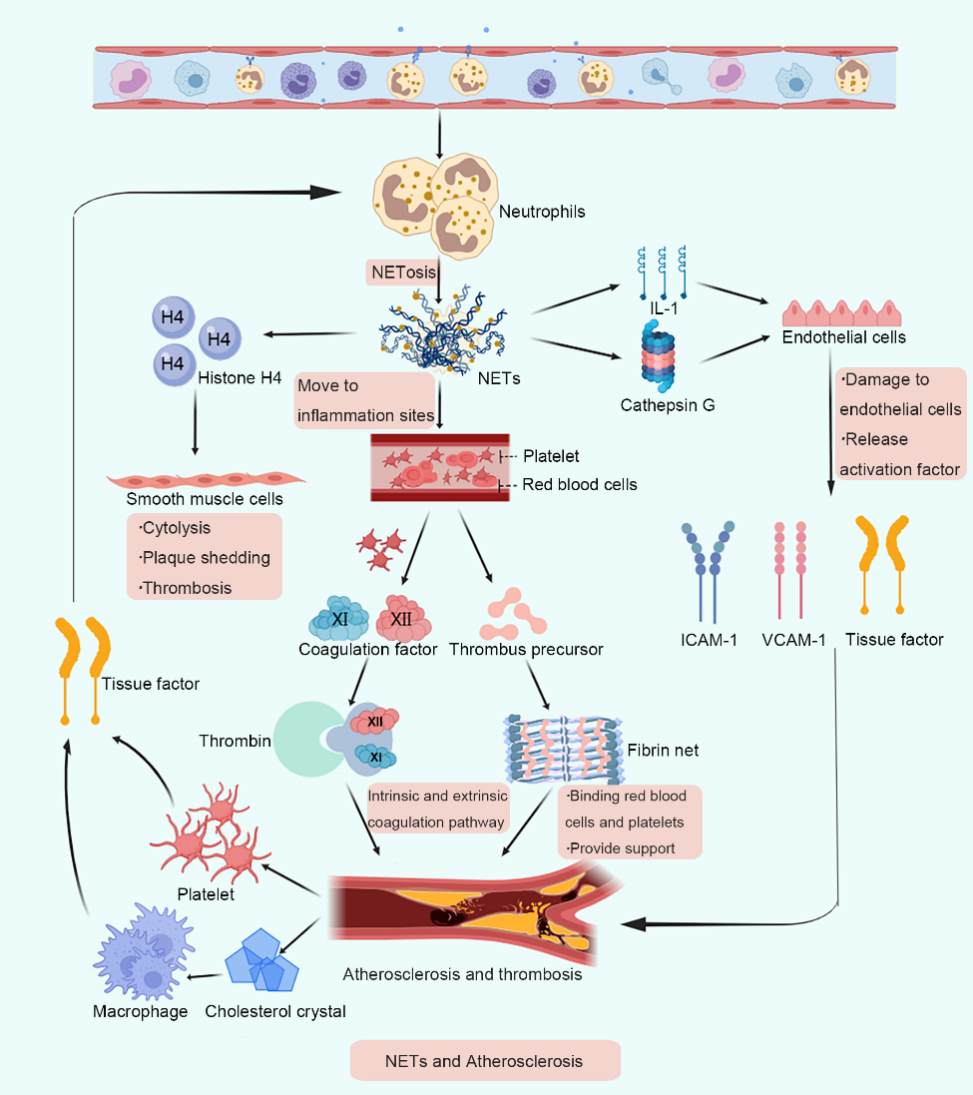
Schematic illustration of molecular mechanisms of neutrophil extracellular traps (NETs) in atherosclerosis. When the inducer stimulates neutrophils, they are activated to produce NETs, which participate in the formation of atherosclerosis through the expression of related molecules. Additionally, NETs promote the adhesion and aggregation of platelets and the binding of red blood cells through two coagulation pathways, providing a physical scaffold for thrombus growth. Furthermore, NETs also damage the endothelium through the joint action of IL-1 and cathepsin G, thereby promoting the expression of ICAM-1, VCAM-1 and tissue factors, participating in the formation of thrombus and causing the lysis of smooth muscle cells through the exogenous histone H4 and aiding in the easy sloughing of the plaque

Additionally, NETs present associated prothrombotic molecules that can directly bind to fibrinogen and accelerate fibrin deposition, which forms a fibrin network that acts as a scaffold for capturing platelets and red blood cells [47]. Additionally, neutrophil elastase can bind to NETs, aiding thrombin in promoting fibrin formation in the presence of platelets and leading to thrombus formation. Furthermore, the degradation of histones with heparin could lead to the increased instability of NETs [17]. Therefore, it can be concluded that NETs maintain thrombus stability (Fig. 3).

### NETs and MI

**Fig. 4:**
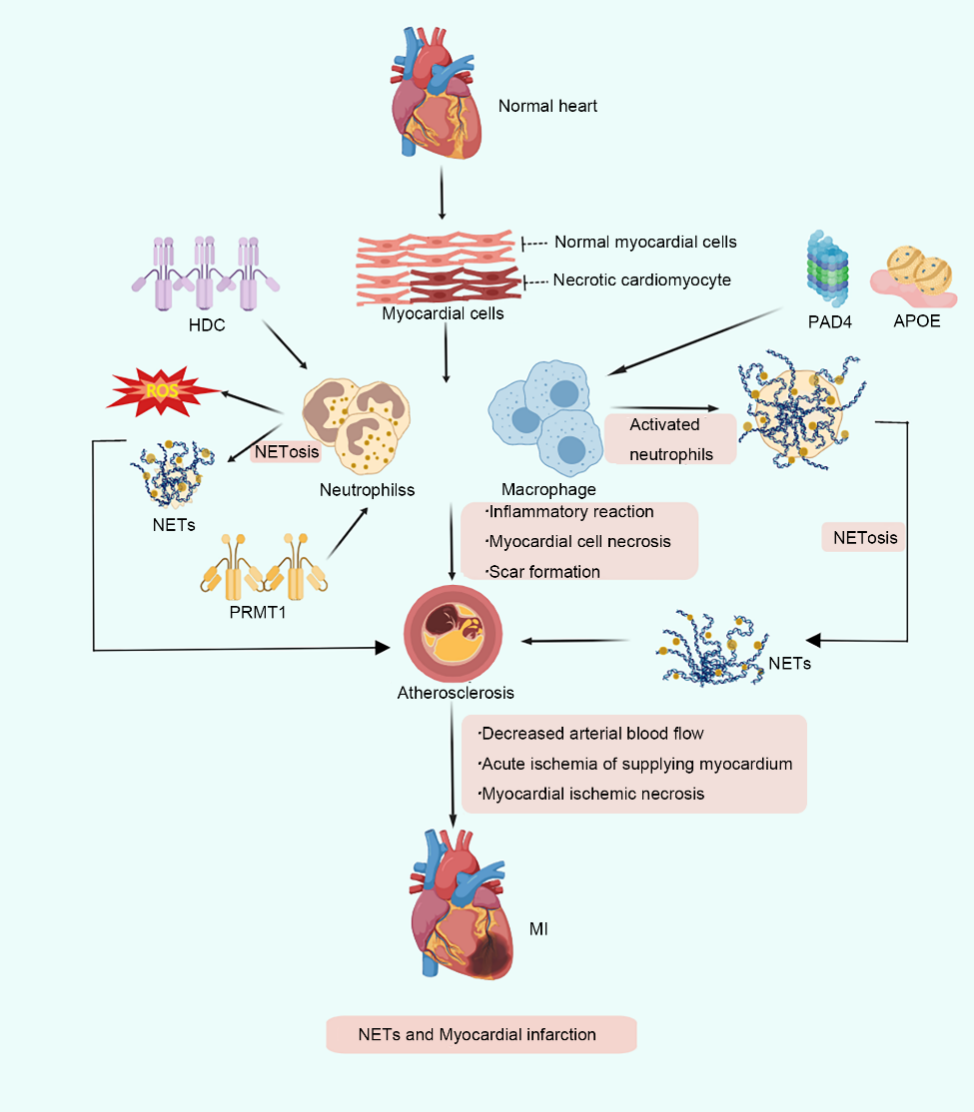
Schematic illustration of the molecular mechanisms of neutrophil extracellular traps (NETs) in myocardial infarction (MI). Cardiac myocyte necrosis in the inflammatory site, HDC and PRMTI act as upstream regulatory signals and directly stimulate neutrophils and promote NETs expression. Additionally, PAD4 and APOE regulate the polarisation of macrophages, indirectly activating neutrophils and promoting the expression of NETs. Furthermore, NETs and inflammatory cells work together on myocardial cells, which damage myocardial cells and induce MI through continuous processes of inflammatory reaction, myocardial cell necrosis and scar formation

MI refers to the ischemic necrosis of the myocardium, wherein the blood flow of the coronary artery is sharply reduced or interrupted at the time of the lesion. This leads to the corresponding supplied myocardium being severely and persistently acutely ischemic and eventually to myocardial ischemic necrosis. Numerous studies have demonstrated that MI is a complex_2_ continuous process, which comprises inflammatory responses, cardiomyocyte necrosis and scar formation. The interaction of the aforementioned factors determines the final disease condition and prognosis of patients with MI. Studies have shown that NETs play a deleterious role in cardiovascular disease [50]. NETs formation not only provides a scaffold for thrombus formation but also aggravates endothelial cell damage, which could be a significant contributor to the development of MI. Furthermore, histidine decarboxylase (HDC), protein arginine methyltransferase 1 (PRMT), peptidyl arginine deaminase 4 (PAD4) and apolipoprotein E (APOE) act as upstream regulatory signals of NETs and exert damaging effects in cardiomyocytes (Fig. 4).

#### HDC exacerbates MI via NETs

HDC is a type of amino acid decarboxylase that mainly catalyzes the decarboxylation of histidine to produce histamine. Regarding MI, studies have found that HDC is associated with ROS and myeloid cell differentiation. Zhang et al. investigated the effect of HDC on ROS production in neutrophils with PMA treatment and observed that the level of ROS produced by neutrophils in the absence of HDC was significantly higher than that in the HDC group, irrespective of PMA treatment [51] Moreover, previous studies have demonstrated that excess ROS promotes NET production, thus highlighting that HDC plays a crucial role in regulating neutrophils function, especially cellular activity. Additionally, NETs isolated from HDC bone marrow were co-cultured with cardiomyocytes under hypoxic conditions for 24 h after PMA activation and revealed that HDC-deficient neutrophils caused more cardiomyocyte-related death than controls. Thus a positive correlation between NETs and cardiomyocyte death was observed. It is also speculated that HDC deficiency in neutrophils would increase cardiomyocyte death through NETs with cardiac fibroblast proliferation and migration, thereby aggravating MI.

#### PRMT1 exacerbates MI via NETs

PRMT is a group of enzymes that catalyze the methylation modification of arginine in proteins, mainly on histone H4, which is involved in gene transcription regulation, protein stability regulation, DNA damage signal and other responses. The inhibition of PRMT1 transcription by histamine significantly reduced myocardial fibrosis in HDC-deficient mice and rescued myocardial injury due to MI. Thus, PRMT1 mediates ROS enhancement and NETs generation due to HDC deficiency in neutrophils. In conclusion, neutrophils are recruited to the ischemic injury site of the myocardium when MI occurs, wherein point HDC deficiency leads to attenuated neutrophil adhesion but enhanced migration. Furthermore, PRMT1 increases the production of ROS and NETs through the H1R-SWI/SNF-PRMT1-ROS pathway [51], which in turn promotes cardiomyocyte death and cardiac fibroblast proliferation, ultimately leading to the aggravation of MI.

#### PAD4 exacerbates MI via NETs

During MI, the innate immune response and rapid recruitment of albumin play a critical role in regulating inflammation and subsequent healing. PAD4 mainly relies on calcium ions to catalyze the conversion of arginine residues to citrulline residues in target proteins, also known as citrullination. Studies have shown that PAD4 is mainly involved in chromosome decondensation during the release of NETs from activated neutrophils [52]. At sites of inflammatory injury, in addition to recruiting neutrophils to release NETs, the ischemic site rapidly recruits monocyte macrophages. In the early stage of inflammation, monocytes are polarized into M1-type cells and gradually replaced by M2-type cells, which promote the resolution of inflammation and tissue remodelling and thereby induce beneficial healing effects on the injured myocardium [52, 53]. Additionally, by stimulating bone marrow-derived macrophages in wild-type (WT) mice with NETs, Kaveh et al. showed that NETs in the presence of INF-δ and LPS and under hypoxic conditions significantly inhibit IL-6 and TNF-α expression but can upregulate IL-10 levels. Therefore, NETs are speculated to drive macrophage polarization toward an anti-inflammatory phenotype. In addition, NETs can also form autoantigens that induce certain diseases, for example, in systemic lupus erythematosus (SLE), NETs can activate plasmacytoid dendritic cells (pDCs) and trigger type I interferon (IFN) production and drive autoimmune pathology [54, 55]. Parackova et al. [56] determined that NETs can be used in the development of diabetes mellitus by stimulating with PMA NETs from type 1 diabetes mellitus (T1D) patients and NETs fragments isolated from peripheral blood of healthy control donors, cleavage of NETs with a restriction endonuclease mixture, and co-culture and further compositional analysis using fractions consisting of large DNA fragments and NETs-associated proteins, showed that the presence of NETs induces IFN-γ-producing T cells in vitro and activates T cells toward IFN-γ-producing CD4 and CD8 polarization. Therefore, we speculate that NETs may exacerbate inflammation by inducing Th1 cell polarization, triggering T cell-mediated immune responses, and directly or indirectly regulating inflammatory cytokines. However, PAD4-deficient mice were unable to release NETs but produce higher levels of ROS and display higher levels of cfDNA, cTNT and pro-inflammatory mediators in the post-MI period, wherein cfDNA was widely considered to be a consequence of MI-associated cell death and neutrophil activation [47]. Furthermore, Zhou et al. [57] reported that NETs could activate macrophages to constantly secrete inflammatory cytokines, thereby promoting the inflammatory response to a certain extent and further aggravating the occurrence and development of diseases. Additionally, cfDNA was observed to increase the expression of IL-10 in cells, thus highlighting the role of NETs in further acting on the myocardial ischemic site and aggravating MI.

#### APOE exacerbates MI via NETs

APOE is a polymorphic protein involved in the metabolism of lipoproteins. The APOE genes can regulate many biological functions and participate in various responses. Moreover, the APOE genotype-phenotype correlates with the occurrence of MI [58]. Zhou et al. [59] established an MI model by performing permanent coronary artery ligation surgery on APOE^−/−^ and WT mice. The results of TTC staining showed that APOE^−/−^ mice had larger infarcts than WT mice, and serum assays revealed that APOE^−/−^ mice had significantly higher serum cTnI and CK-MB levels. Thus, these findings indicate that APOE deficiency can aggravate ischemic injury after MI. Additionally, APOE deficiency could exacerbate the activation of neutrophils after MI through an NADPH oxidase-ROS-dependent pathway, promoting the formation of NETs, acting on the site of myocardial ischemic injury, promoting the proliferation of fibroblasts and leading to the exacerbation of myocardial injury in MI. Furthermore, PAD4 could provide a novel therapeutic strategy for protecting the myocardium by aiding in the inhibition of NETs formation via NADPH oxidase inhibition. Therefore, there exists a clear correlation between NETs and poor prognosis of MI. Refining the role and mechanism of NETs in MI and further validating the feasibility of NETs in the treatment of MI has promising diagnostic and therapeutic potential.

### NETs and heart failure

**Fig. 5:**
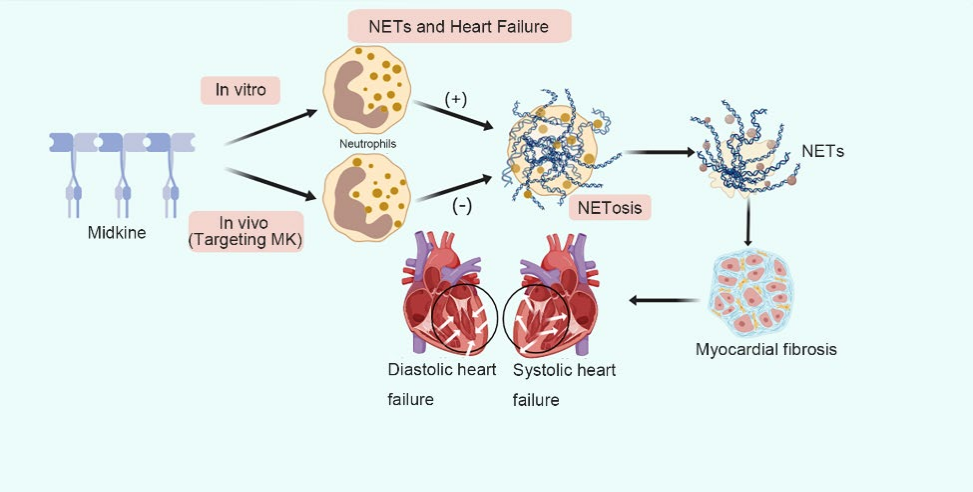
Schematic illustration of molecular mechanisms of neutrophil extracellular traps (NETs) in heart failure. Midkine has a significant regulatory effect on NETs. It can promote the formation of NETs in vitro, inhibit the formation of NETs and reduce the infiltration of neutrophils in vivo. Furthermore, NETs induce myocardial fibrosis and cause cardiac systolic or diastolic dysfunction, which is manifested as systolic or diastolic heart failure

Heart failure refers to a clinical syndrome wherein the sizeable venous return volume cannot be fully discharged out of the heart due to cardiac systolic and/or diastolic dysfunction, resulting in blood stasis in the venous system and insufficient blood perfusion in the arterial system. This syndrome is considered the terminal stage in the development of all cardiovascular diseases. Midkine (MK), which mainly mediates the formation of NETs in vitro, can attenuate the formation of NETs and neutrophil infiltration in vivo. Using experimental autoimmune myocarditis (EAM) mouse model to induce the generation of NETs, Ludwig T et al. [60] observed that the treatment of mice with DNase or protein arginine deamination inhibitor induced leukocyte infiltration in EAM. Furthermore, compared with the untreated model, NETs promoted myocardial inflammation during EAM. This illustrated that targeting MK could reduce neutrophil infiltration and NETs formation in the myocardium. Additionally, the inhibition of MK has also been reported to reduce the infiltration of neutrophils in cardiac tissue and release NETs, thereby reducing myocardial fibrosis [60]. However, the exact mechanisms by which MK promotes the recruitment of NETs and the induction of heart failure by NETs remains unclear. Therefore, serum MK level may have the potential as a diagnostic indicator in patients with heart failure, reflecting the severity of heart failure and the degree of impairment of cardiac function. Thus, targeting MK could reduce neutrophil infiltration and NETs formation in the inflamed myocardium. Furthermore, the inhibition of MK or NETs is a potential therapeutic target for patients with heart failure (Fig. 5).

## NETs as therapeutic targets for ACS

Recent studies have revealed that NETs play an essential role in cardiovascular diseases. NETs accelerate the development of ACS through several pathways, with the individual components acting as autoantigens, interacting with multiple cell types, activating inflammasomes and accelerating the atherosclerotic process [17]. Previous studies also report that the level of NETs was negatively correlated with ST-segment regression and positively correlated with infarct size in patients with ST-segment elevation myocardial infarction [61, 62]. Zhou et al. [59] reported that the inhibition of NETs production attenuated the extent of myocardial injury in APOE^−/−^ mice with reduced infarct size and neutrophil infiltration. Therefore, NETs burden could be considered a predictor of infarct size in ST-segment elevation coronary syndrome. In patients with heart failure, the MK level increases with the heart failure index, thus the serum MK level reflects the severity of heart failure and the course of the disease in patients [63, 64]. Numerous data also suggest that the number of neutrophils and the formation of NETs regulate the early progression of cardiovascular disease. Thus, NETs could provide a potential action target for the clinical treatment of ACS. However, correlation does not necessarily imply causation, and since inflammatory responses precede the formation of NETs, inhibition of NETs may not serve as prophylactic treatment for ACS episodes. In addition, long-term inhibition of inflammation may have other complications, such as possible inhibition of M2 macrophage induction, which may affect tissue repair and regeneration after inflammatory injury. However, in some cases, inhibition of NETs may be beneficial in reducing the inflammatory response and preventing further injury, so the effectiveness of this approach may depend on the stage of the disease and the severity of the inflammatory process, and the optimal use of NETs inhibition as a treatment for ACS may need to focus on the patient’s disease stage, comorbidities, and other factors. Liu et al. [65–67] demonstrated that neutrophil granulocytes generate NETs during the acute inflammatory response phase of the disease, and high NET levels polarize macrophages to the M1 phenotype; therefore, it is speculated that NETs may play an important role in the interaction between neutrophils and macrophages during the acute phase, and that NETs inhibition during the acute inflammatory response phase could be used to treat ACS.

## Potential therapeutics targeting NETs

An increasing number of studies have found that the formation of neutrophil activation-associated NETs is closely related to the pathogenesis of ACS. The study of NETs and their relevance to ACS may be a therapeutic target for preventing and treating ACS [61]. Several practical therapeutic approaches will be discussed below (Table 1).

**Table 1:** Potential Approaches for Targeting NETs for ACS

Goal	Drugs	Strategies	Action	Reference	
PAD4	CL-amidine	PAD4 Inhibitor	Blocking histone guanylation in NETosis	[70–72]	
DNA matrixes	DNase I	DNA degradation	Degradation of the DNA composition of NETs	[73, 74]	
NADPH/ROS	N-acetylcysteine, 5-Aminosalicylic acid, Kaem	Antioxidant	Inhibition of NADPH oxidase-ROS-dependent pathway	[77]	
Citrulline protein	Anti citrulline protein antibody	Antibodies NET-inhibiting	Inhibitting the formation and uptake of NETs by macrophages	[92]	
Platelet	Heparin Aspirin	Anticoagulant	Selectiveing inhibition of platelet aggregation and NETs formation	[81, 82]	
Inflammatory mediators	TLR antagonists,CXCR blockers, Homocysteine (HHcy), Colchicine	Inflammatory mediator receptor blockers	Acting by inhibiting inflammatory vesicle activity to reduce the load of NETs in recent myocardial infarction or stroke	[86–91]	

### PAD4 inhibitor

PAD4 is a protease that catalyzes the guanylation of arginine residues and is involved in NETosis. CL-amidine, a PAD4 mimetic peptide, competitively binds to PAD4 and inhibits its action [68]. Therefore, inhibition of PAD4 by drugs such as chloro-amidine is a possible target for treating NET-related diseases. CL-amidine exerts its inhibitory effect mainly by covalently modifying conserved cysteine residues in the active site of PAD645 and is an irreversible PAD4 inhibitor that is highly selective for PAD4 [69]. Kinght et al. [70] found that the PAD4 inhibitor chloro-amidine blocked the formation of NETs in APOE-/- mice fed a high-fat diet, reduced atherosclerosis lesion size, and delayed post-injury thrombosis, leading to the treatment of ACS. In acute carotid injury experiments, Franck et al. [71] found that PAD4 deficiency protected mice from plaque erosion. Furthermore, Du et al. [72] found that myocardial ischemic injury significantly increased PAD4 expression activity and that direct inhibition of PAD4 protected the myocardium from inflammation. PAD4 inhibitors prevented the formation of NETs in plaques, reduced the number of endothelial macrophages, decreased neutrophil recruitment to the vessel wall, and reduced levels of inflammatory mediators, ultimately significantly reducing atherosclerotic plaque formation and thrombosis and decreasing the risk of myocardial infarction [70].

### DNase I

DNase I is a deoxyribonuclease that lyses the DNA component of NETs and has been used as a pharmacological intervention to treat various diseases. Since DNA is a significant component of NETs, inhibition of NETs formation with DNase I is highly likely for the treatment of ACS. DNase I treatment is an effective therapeutic tool for neutrophil-derived diseases caused by NETs. Warnatsch et al. [73] demonstrated that systemic injection of DNase I reduced the size of atherosclerotic plaques in mice and decreased the number of NETs and the number of inflammatory cells. In addition, Frank et al. [74] administered DNase I in a PAD4-/- mouse model and found reduced surface erosion of endothelial cells and an increase in surviving endothelial cells while limiting the recruitment of neutrophils. It has been shown that DNase I can partially lyse NETs. However, whether the degradation of NETs by DNase I leads to the breakdown of histones with coagulant activity, which in turn increases the risk of thrombosis, needs further investigation [75].

### Antioxidant

The NADPH oxidase-derived active enzyme ROS is essential for the formation of NETs. ROS has been shown to induce different levels of DNA damage and activate neutrophil elastase, which contributes to chromatin cleavage and NETs formation [76]. Through a PAM-induced ROS model, Zeng et al. [77] found that antioxidants inhibit ROS in activated neutrophils production and dsDNA release and that the inhibition of NETs by antioxidants was achieved by inhibiting ROS if the cells were pretreated with NADPH inhibitors, which did not result in dsDNA release. It was shown that antioxidant substances that significantly inhibit ROS release from neutrophils likewise inhibit the formation of ROS-dependent NETs, suggesting a clear correlation between the two [78].

### Anticoagulant

Heparin is a multifunctional glycosaminoglycan with anticoagulant activity and antithrombotic effects that inhibits the activation of neutrophils to produce NETs while promoting the breakdown of NETs, releasing histones from the chromatin backbone and destabilizing DNA, thereby disrupting the coagulation effect of NETs and preventing thrombosis [79–81]. Angelo et al. [82] showed that treating NET-related diseases with heparin depends in part on the induction of a “refractory” state, leading to a decrease in the stimulation of the inflammatory response and a decrease in the release of NETs. In addition, NETs can induce endothelial cell damage. The degree of damage is positively correlated with the concentration of NETs. Heparin can reduce NET-induced endothelial damage, which may be a potential mechanism for the protective effect of heparin on ACS [83].

### Inflammatory mediator receptor blockers

Colchicine, a novel therapeutic agent, has demonstrated its safety and efficacy by inhibiting the inflammatory cascade response in patients with extracranial or intracranial atherosclerosis or slight arteriosclerosis, thereby reducing vascular events [84, 85]. The CALCOT trial [86–88] showed that compared with a placebo, low doses of colchicine significantly reduced the risk of recurrence in patients with ischemic cardiovascular disease risk. The EQUALTRCT trial [89–91] demonstrated that colchicine inhibits the activity of inflammatory vesicles, which can reduce the load of NETs in cardiovascular disease and effectively prevent the development of ACS.

## Conclusions and future directions

Recently, the incidence of cardiovascular disease has been increasing worldwide, with ACS posing a significant threat to human health. As an important immune cell, neutrophils are vital to the study of cardiovascular diseases. Notably, NETs have become a popular research direction in the search for therapeutic targets in cardiovascular diseases. Since their discovery in 2004, various experimental and clinical literature has confirmed the role of NETs in various cardiovascular diseases and their correlation with the poor prognosis of diseases. However, the role and mechanism of NETs in various diseases remain to be fully elucidated. Additionally, controversies about the translation of NETs in clinical settings remain. Nonetheless, new insights from the study of NETs in cardiovascular diseases are expected to open new avenues for diagnosing and treating of ACS. This review explored the impact of NETs on ACS, identifying that NETs play a novel and essential role in ACS and highlighting its potential as a novel target for the prevention and treatment of ACS.

## Data Availability

Not applicable.
